# Genomic Dissection of Anthracnose (*Colletotrichum sublineolum*) Resistance Response in Sorghum Differential Line SC112-14

**DOI:** 10.1534/g3.120.401121

**Published:** 2020-02-21

**Authors:** Clara M. Cruet-Burgos, Hugo E. Cuevas, Louis K. Prom, Joseph E. Knoll, Lauren R. Stutts, Wilfred Vermerris

**Affiliations:** * USDA-ARS, Tropical Agriculture Research Station, 2200 Pedro Albizu Campos Avenue, Mayagüez, Puerto Rico 00680; † University of Puerto Rico, Department of Biology, Mayagüez, Puerto Rico; ‡ USDA-ARS, Southern Plains Agriculture Research Center, 2881 F & B Road, College Station, Texas 77845; § USDA-ARS, Crop Genetics and Breeding Research, 115 Coastal Way, Tifton, Georgia; ** Graduate Program in Plant Molecular & Cellular Biology, University of Florida, Gainesville, Florida 32610; †† Department of Microbiology & Cell Science, UF Genetics Institute, and Florida Center for Renewable Fuels and Chemicals, Gainesville, Florida 32610

**Keywords:** Anthracnose, Genotype-by-sequencing, Genome mapping, KASP markers, Sorghum

## Abstract

Sorghum production is expanding to warmer and more humid regions where its production is being limited by multiple fungal pathogens. Anthracnose, caused by *Colletotrichum sublineolum*, is one of the major diseases in these regions, where it can cause yield losses of both grain and biomass. In this study, 114 recombinant inbred lines (RILs) derived from resistant sorghum line SC112-14 were evaluated at four distinct geographic locations in the United States for response to anthracnose. A genome scan using a high-density linkage map of 3,838 single nucleotide polymorphisms (SNPs) detected two loci at 5.25 and 1.18 Mb on chromosomes 5 and 6, respectively, that explain up to 59% and 44% of the observed phenotypic variation. A bin-mapping approach using a subset of 31 highly informative RILs was employed to determine the disease response to inoculation with ten anthracnose pathotypes in the greenhouse. A genome scan showed that the 5.25 Mb region on chromosome 5 is associated with a resistance response to nine pathotypes. Five SNP markers were developed and used to fine map the locus on chromosome 5 by evaluating 1,500 segregating F_2:3_ progenies. Based on the genotypic and phenotypic analyses of 11 recombinants, the locus was narrowed down to a 470-kb genomic region. Following a genome-wide association study based on 574 accessions previously phenotyped and genotyped, the resistance locus was delimited to a 34-kb genomic interval with five candidate genes. All five candidate genes encode proteins associated with plant immune systems, suggesting they may act in synergy in the resistance response.

Sorghum [*Sorghum bicolor* L. (Moench)] is a highly diverse cereal crop used for food, animal feed, fodder, and biofuel production. It is currently the fifth most important cereal crop behind maize (*Zea mays* L.), wheat (*Triticum aestivium* L.), rice (*Oryza sativa* L.), and barley (*Hordeum vulgare* L.), with an annual global production of 61.4 Mt produced on 42.1 million hectares (FAOSTAT 2017). Sorghum's predominant mode of reproduction is via self-pollination, with outcrossing rates that vary from 5 to 40% under field conditions (Barnaud *et al.* 2008). It has lower water and nutrient requirements than many other crops, making it resilient and suitable for a wide range of environments. Its production is currently primarily associated with hot and dry regions in tropical, sub-tropical and temperate regions. Given the versatility of the crop, its production is expanding to warm and humid regions where its profitability is being limited by biotic constraints.

Anthracnose, caused by the fungal pathogen *Colletotrichum sublineolum* P. Henn., in Kabat and Bubák, is the major disease of sorghum in the Southeastern United States and the Caribbean, where sorghum acreage has been expanding rapidly in the last decade (USDA 2017). *C. sublineolum* affects all above-ground tissues, causing yield losses of both grain and biomass of up to 50% in highly susceptible cultivars (Thakur and Mathur 2000). Multiple pathotypes have been identified based on their virulence against eighteen sorghum differential lines (Prom *et al.* 2012), but this virulence was neither associated with geographic regions or phylogeny. The disease is usually controlled by crop rotation and fungicide application (Gwary and Asala 2006; Resende *et al.* 2013). The most cost-effective and environmentally benign way to control anthracnose, however, is through incorporation of resistance genes or host plant resistance.

Several sources of anthracnose resistance have been identified in tropical and temperate-adapted germplasm. The screening of the U.S. National Plant Germplasm System (NPGS) sorghum germplasm collection resulted in multiple sources of resistance being identified (Prom and Erpelding 2009; Erpelding 2011; Prom *et al.* 2011; Erpelding 2012; Cuevas *et al.* 2014b; Cuevas *et al.* 2016). Most of these resistance sources are, however, tropical germplasm that cannot be integrated into U.S. sorghum breeding programs without conversion through introgression of photoperiod insensitivity using dwarfing and early maturity genes (Thurber *et al.* 2013). The sorghum association panel (SAP) is comprised of 149 U.S. breeding lines and 228 lines adapted to temperate regions and was assembled to capture the majority of genetic diversity present in sorghum germplasm and breeding programs (Casa *et al.* 2008). Among 335 accessions from the SAP evaluated against pathotypes from Puerto Rico, Texas and Georgia, 37 were resistant to all tested pathotypes of anthracnose (Cuevas *et al.* 2018; Prom *et al.* 2019). Unfortunately, the lack of information on the genetic control of resistance in these sources has limited their use in breeding programs.

Understanding the inheritance of anthracnose resistance is imperative for its adequate use and durable control of disease. In the U.S., some resistance sources have been studied using biparental mapping approaches to identify genomic regions associated with resistance. Anthracnose tolerance observed in breeding line Sureño was associated with a quantitative trait locus (QTL) on chromosome 6 that also provides resistance to other pathogens (Klein *et al.* 2001). Three studies identified anthracnose resistance loci on chromosome 9 in sorghum lines BS04/05 (Biruma *et al.* 2012), Bk7 (Felderhoff *et al.* 2016) and SC155-14E (Patil *et al.* 2017). Biruma *et al.* (2012) showed that the *R*-genes *Cs1A* (long arm of chromosome 9) and *Cs2A* (short arm of chromosome 9) were the primary sources of resistance in BS04/05. The resistance locus from Bk7 reported by Felderhoff *et al.* (2016) did not include *Cs2A*, but it is not yet clear whether the source of the resistance in this study is the same as the one identified by Patil *et al.* (2017) in SC414-12E.

The distal region of chromosome 5 has been associated with the anthracnose resistance response in sorghum lines SC748-5 (Ramasamy *et al.* 2009; Burrell *et al.* 2015), SC112-14 (Cuevas *et al.* 2014a) and SC414-12E (Patil *et al.* 2017). Comparative analysis indicated that the resistance loci in SC112-14 and SC414-12E overlapped, while the locus in SC748-5 was in a distinct physical location. These studies also revealed the presence of other smaller-effect loci. Fine mapping the loci on chromosomes 5 and 9 to individual gene(s) is necessary to make the most efficient use of maker-assisted selection in breeding programs.

Genome-wide association studies (GWAS) for anthracnose resistance response using the SAP and a core-set of the NPGS Ethiopian collections identified candidate genes within QTL intervals previously identified on chromosomes 5 and 9. Based on the anthracnose resistance response in the SAP observed in Puerto Rico and Georgia (U.S.A.), three loci were identified within a 12-Mb region on chromosome 5 that may match the loci identified in SC112-14, SC414-12E, and SC748-5 (Cuevas *et al.* 2018). Nevertheless, these three loci only explained 56% of the observed phenotypic variation, implying the presence of other resistance sources in the panel. Subsequent analysis of the SAP limited to resistance response against Texas pathotypes identified multiple loci across the genome, suggesting complex genetic control (Prom *et al.* 2019). Likewise, a resistance locus at the distal end of the short arm of chromosome 9, that explained up to 31% of the anthracnose resistance variation present in NPGS Ethiopian core set, could be related to the loci identified in Bk7 and/or SC155-14E (Cuevas *et al.* 2019). Even though both GWAS studies led to the identification of candidate genes within the QTL interval, confirmation with large segregating progenies or via functional genomic analysis will be necessary.

Even though previous research identified the existence of multiple anthracnose resistance sources in temperate adapted germplasm that are accessible for sorghum breeding programs, inheritance studies are limited to only a few resistance sources, while high-throughput molecular markers to enable introgression into commercial varieties are absent. In the current study, 114 recombinant inbred lines (RILs) derived from anthracnose-resistant sorghum differential line SC112-14 were used to: 1) determine its resistance response against pathotypes from four locations; 2) identify and confirm resistance loci based on high-density linkage maps; 3) evaluate resistance response against 10 different *C. sublineolum* pathotypes using the bin-mapping approach; 4) fine-map a resistance locus on chromosome 5 to candidate genes using high-throughput molecular markers.

## Material and methods

### Germplasm and field experiments

The recombinant inbred lines (RILs; F_5:6_) used in this study were obtained by using the single seed descent method from the cross of the sorghum anthracnose resistant line SC112-14 (PI533918) and susceptible PI609251 (Cuevas *et al.* 2014a). The temperate adapted line SC112-14 (No. 9 Gambela) is originally from Ethiopia, belongs to the Durra race working group Zerazera and has been used to establish *C. sublineolum* pathotypes both in Brazil and the United States (Valèrio *et al.* 2005; Prom *et al.* 2012). The line PI609251 (CIRAD 15) is originally from Mali, belongs to the Caudatum race working group Caudatum-kafir, and was selected because of its broad spectrum of susceptibility to anthracnose (Prom *et al.* 2012).

A total of 114 RILs and parental lines were planted during 2016 at the research farm of the USDA-ARS Tropical Agriculture Research Station in Isabela, Puerto Rico, U.S.A. (18°0.28’18.4”N, 67°02’37.2”W), at the Black Shank Farm of the University of Georgia in Tifton, Georgia, U.S.A. (31°29’58.9”N, 83°32’53.8”W), at the Research Farm of the Texas A&M University at College Station, Texas, U.S.A. (30°31’55.5”N, 96°25’23.7”W), and at the University of Florida Suwannee Valley Agricultural Research and Education Center near Live Oak, Florida, U.S.A. (30°18’47.8”N, 82°54’07.8”W). The experimental design at all locations was a randomized complete block design (RCBD) consisting of two blocks with single-row plots of 3.1 m in length with 10-20 plants and 0.9 m between rows.

### Anthracnose resistance

The 114 RILs and their parental lines were evaluated in the four locations listed above, where anthracnose is an endemic disease. Even though *C. sublineolum* is naturally present at each location, several plants per row were manually inoculated to ensure consistent symptoms in case of non-uniform distribution of inoculum in the field. This was accomplished by culturing three to five *C. sublineolum* isolates from each location on potato-dextrose agar (PDA), followed by the inoculation and colonization of autoclaved sorghum seeds. Approximately ten *C. sublineolum*-colonized seeds were placed into the leaf whorl of 30-45 day-old-plants. The anthracnose resistance responses of the lines were determined approximately 30-45 days after flowering (hard-dough stage to physiological maturity) using the scale described by Prom *et al.* (2009), which has been proven successful for identifying anthracnose resistance loci (Ramasamy *et al.* 2009; Felderhoff *et al.* 2016; Cuevas *et al.* 2018; Cuevas *et al.* 2019). Each single-row plot was visually inspected for presence of anthracnose disease and the most infected plant was used to determine the disease score based on a 1-5 scale, where 1 = no symptoms or chlorotic flecks in the plot; 2 = hypersensitive reaction, but no acervuli present in the plot; 3 = infected bottom leaves with acervuli formation in at least one plant within the plot; 4 = necrotic lesions with acervuli observed on bottom leaves and spreading to middle leaves in at least one plant within the plot; and 5 = most leaves necrotic due to infection, including infection of the flag leaf in at least one plant within the plot. The basis for this rating is that the plant displaying the most severe disease phenotype represents the actual susceptibility of the line, while the variation within the plot is associated with the distribution of the infection in the field.

The anthracnose resistance responses of the RILs were first categorized as resistant (score ≤2.0) or susceptible (score >2.0) and subjected to a χ^2^ test (SAS 9.4, SAS Institute, Cary, NC) against the 1:1 expected phenotypic ratio due to the segregation of one resistance locus. In parallel, the anthracnose resistance responses of the RILs across locations were combined and subjected to analysis of variance using the *PROC MIXED COVTEST* method *type 3* procedure of SAS 9.4 (SAS Institute, Cary, NC). The locations were considered fixed, whereas RILs and blocks were treated as random effects. The anthracnose resistance response across and within locations was estimated based on least square means. The narrow-sense heritability (*h^2^*) across locations was estimated using the formula:

$$h^{2}=\frac{\sigma_{g}^{2}}{\sigma_{g}^{2}+\frac{\sigma_{G\times L}^{2}}{l}+\frac{\sigma_{e}^{2}}{rl}},$$

where $\sigma_{g}^{2}$, $\sigma_{G\times L}^{2}$, $\sigma_{e}^{2}$ refer to the genotypic (RILs), genotype-by-location, and error variances, respectively, while *r* and *l* are the number of block and locations, respectively (Bernardo 2002).

### High-density linkage map

A genotype-by-sequencing (GBS) library was prepared and sequenced at the Institute for Genomic Diversity (Cornell University, Ithaca, New York, U.S.A) using the restriction enzyme *Ape*K1 for digestion (Elshire *et al.* 2011). The GBS library was sequenced in one-quarter of a lane of an Illumina HiSeq 2500, followed by SNP calling using TASSEL 5 GBS v2 Pipeline (Glaubitz *et al.* 2014) and the most recent version of the BTx623 sorghum genome (version 3.1; www.phytozome.net, accessed June 26, 2019). Raw genotypes (42,128 SNPs) were filtered according to minor allele frequency (MAFs) (>0.40) and missing data (<0.50). After filtering 9,711 SNPs were retained and missing data were imputed using Beagle 4.1 (Browning and Browning 2016). The SNPs that were imputed with a probability call of <0.80 and heterozygous were retained as missing data. The imputed genotype data were filtered for MAFs >0.40, missing data (<0.80), segregation distortion against a 1:1 expected ratio [χ^2^ *P*(value) <0.05], which resulted in a total of 7,663 SNPs.

An initial linkage map was constructed with MSTmap software (Wu *et al.* 2008) using a LOD criterion >10, Kosambi mapping distance, and genotyping error detection. The resulting map was visually inspected to identify and remove SNPs with unlikely double recombination events within each bin. A total of 3,838 high-quality SNPs (*i.e.*, missing data <9%, 1:1 segregation ratio, and lowest number of genotyping errors) was retained and ordered for a second time with MSTmap software as previous described (Wu *et al.* 2008). The recombination linkage map was compared against the most recent version of the BTx623 sorghum genome (version 3.1; www.phytozome.net, accessed June 26, 2019) to confirm their collinearity (Supplemental Figure 1).

### QTL analysis

Composite interval mapping (CIM) was conducted in QTLCarthographer v2.5 (Wang *et al.* 2012) to identify anthracnose resistance loci across and within location. The backward regression method with a window size of 10 cM and five markers to reduce background effects were established to scan the genome with a walking speed of 1 cM. The thresholds for determining a statistically significant QTL were calculated with 1,000 permutations for experiment-wise error rates of α = 0.05.

### Bin mapping

The high-density genetic map of RILs and the Mappop v.1.0 software (Vision *et al.* 2000) were used to select a subset of 31 highly informative RILs to evaluate ten pathotypes from Texas, Georgia, Puerto Rico and Arkansas in the greenhouse. These 31 RILs were chosen using the *SAMPLEMAX* command with a 0.30 fraction of the whole population in Mappop v.1.0. (Vision *et al.* 2000). This sample was determined suitable for genome mapping with a minimal loss of precision. These pathotypes were selected based on previous genetic characterization of the genetic diversity of *C. sublineolum* (Prom *et al.* 2012).

The 31 RILs, parental lines, and the reference lines BTx623 (susceptible), TAM428 (susceptible), and SC748-5 (resistant) were planted in the greenhouse facilities of the Southern Plains Agriculture Research Center, College Station, Texas, USA. The experimental design for each pathotype (*i.e.*, 10 separate experiments) was a randomized complete block design (RCBD) consisting of three blocks each with 36 tall tree pots (11.4 liter) and four plants per tree pot. Sorghum plants were inoculated by placing approximately ten *C. sublineolum*-colonized seeds in the whorl when the plants had reached the 8-10 leaf stage and spraying with a 3-5 mL conidial suspension (10^6^ conidia mL^−1^). Plants were misted for 30 s at 45-min intervals for 8 h per day to provide an adequately humid environment for disease development. The anthracnose resistance responses of the RILs was determined approximately 30-45 days after inoculation and rated as resistant or susceptible based on the presence or absence of acervuli formation on inoculated leaves.

A single-marker analysis was conducted in Tassel 5.0 (Glaubitz *et al.* 2014) using the binary data (*i.e.*, resistant or susceptible) to identify anthracnose resistance loci for each pathotype. The thresholds for determining a significant association were calculated with 1,000 permutations for experiment-wise error rates of α = 0.05 and 0.001. Manhattan plots were visualized with the R package qqman (Turner 2018).

### Fine-mapping of anthracnose resistance locus

A QTL located in the distal region of chromosome 5 (62.34 - 67.59 Mb) was fine-mapped to identify candidate genes. Based on the previous F_2_ genotyping and phenotyping segregation analysis two plants heterozygous in the QTL region and resistant to anthracnose were selected to fine-map the loci (Cuevas *et al.* 2014a). Therefore, the derived F_2:3_ families from these two individuals were segregating for the contrasting alleles in the QTL region. Five SNP markers [four kompetitive allele-specific PCR (KASP) and one insertion:deletion (INDEL)] were developed based on GBS results and genomic sequence analysis of parental lines (Supplementary Table S1 and S2). These five markers divided the locus into four genomic regions of 56, 330, 20, and 449 kb. A total of 1,500 segregating individuals were germinated in the greenhouse and genotyped with the two KASP markers (SNP_64784452 and SNP_65640662) that flank the locus to identify recombination events. Recombinant individuals were genotyped with two additional KASP markers (SNP_64840542 and SNP_65194327), and the INDEL (SNP_65171076) by direct sequencing. Recombinants and parental lines were transplanted at the research farm of the Tropical Agriculture Research Station at Isabela, Puerto Rico, U.S.A. and inoculated as previously described. The anthracnose resistance responses of the recombinant and parental lines were determined approximately 30-45 days after inoculation and rated as resistant or susceptible based on the presence or absence of acervuli formation on inoculates leaves. The Kosambi recombination distance between SNPs and resistance locus were calculated with the R package *OneMap* (Margarido *et al.* 2007).

### Genome-wide association analysis within the anthracnose resistance locus

Three previous GWAS analyses for anthracnose resistance response in the sorghum association panel (SAP) (Cuevas *et al.* 2018; Prom *et al.* 2019), and NPGS Ethiopian collection (Cuevas *et al.* 2019) were combined to conduct an association analysis limited to the 276 SNPs present in the resistance locus on chromosome 5 (65.17 to 65.64 Mb). A total of 280 accessions from SAP have been evaluated against pathotypes from Puerto Rico, Georgia and Texas, U.S.A. of which 35 accessions were found to be resistant across locations. The evaluation of 294 accessions from NPGS Ethiopian collection in Puerto Rico identified 140 anthracnose-resistant accessions. Since the locus on chromosome 5 provides resistance across these three locations, we merged the binary data from these 574 accessions (175 resistant and 399 susceptible; Supplementary Table S5). A multivariate logistic regression model was fitted to the binary data with PLINK (Purcell *et al.* 2007), where the first three principal components were included as covariates to control for population structure and family relatedness. Empirical significance thresholds for the logistic regression were calculated with 1,000 permutations for experiment-wise error rates of *P* = 0.05 [-Log_10_ (*p-value*) = 3.29] and 0.001 [-Log_10_ (*p-value*) = 4.26].

### Data availability

All the data described and used in this manuscript are available as supplemental files, tables, and figures. Table S1 contains the SNP-based (KASP and INDEL) markers information. Table S2 contains the PCR cycle conditions for the KASP markers. Table S3 contains the anthracnose resistance responses of recombinant inbred lines. Table S4 contains the linkage map information based on the 3,838 SNPs. Table S5 contains the anthracnose resistance response of 574 accessions from SAP and NPGS Ethiopian collection. Supplemental material available at figshare: https://doi.org/10.25387/g3.11794347.

## Results

### Anthracnose resistance response in RIL populations

The anthracnose resistance response of RILs across the four locations revealed resistance response dependence on *C. sublineolum* pathotypes (Table 1 and 2; Supplementary Table S3). We observed that of the 109 RILs across locations, 16 were consistently resistant, (≤2.0; no acervuli observed), 50 were consistently susceptible (>2.0; acervuli present), whereas 43 showed a variable resistance response depending on the location. Segregation in Florida, Georgia and Puerto Rico deviated from the expected 1:1 ratio (resistant: susceptible) for a single resistance locus [χ^2^ (*p-value*) <0.05] due to an excess of susceptible lines so that the response observed to the pathogen was considered quantitative in nature. The narrow-sense heritability was estimated to be 0.89, indicating most of the observed variation relates to genetic factors.

**Table 1:** Analysis of variance for anthracnose resistance response of the recombinant inbred line (RILs) population derived from the cross between SC112-14 and PI609251 evaluated at four locations in 2016

	Overall			Florida		Georgia		Texas		Puerto Rico	
Source	df	*p*-value	Source	df	*p*-value	df	*p*-value	df	*p*-value	df	*p*-value
Location	3	**	Block	1	**	1	n.s.	1	n.s.	1	n.s.
Location (Block)	4	n.s.	RIL	105	***	105	***	107	***	108	***
RIL	113	***									
RIL x Location	311	***									

n.s., ** and *** refers to no significance and significant differences at *P* < 0.01 and 0.001, respectively.

**Table 2:** Average and standard deviations of anthracnose resistance response of parental lines and 114 recombinant inbred lines (RILs) derived from the cross between SC112-14 and PI609251 evaluated at four locations in 2016

	Florida		Georgia		Texas		Puerto Rico		Overall	
	X ± SD.		X ± SD.		X ± SD.		X ± SD.		X ± SD.	
SC112-14	2.0 ± 0.0		2.0 ± 0.0		2.0 ± 0.0		2.0 ± 0.0		2.0 ± 0.0	
PI 609251	4.0 ± 0.0		4.0 ± 0.0		3.1 ± 0.3		4.8 ± 0.5		3.7 ± 0.7	
RILs	3.1 ± 1.1		3.2 ± 1.1		2.7 ± 0.7		3.5 ± 1.2		3.1 ± 0.9	
	RILs	χ^2^	RILs	χ^2^	RILs	χ^2^	RILs	χ^2^	RILs	χ^2^
Resistant	41	0.02	39	0.01	45	0.08	33	<0.001	18	<0.001
Susceptible	65		67		63		78		96	

Anthracnose resistance response based on 1-5 scale (Prom *et al.* 2011), where 1-2 are considered resistant and 3-5 susceptible. χ^2^ refer to chi-square test against the 1:1 expected segregation ratio.

### Genome mapping of anthracnose resistance response

The ability to identify QTL is determined by the size of the mapping population and the resolution of the linkage map (Vales *et al.* 2005). The linkage map was based on 3,838 SNP tags and 844 recombination bins and was collinear to the BTx623 genome reference except in some centromeric regions (Supplementary Table S4 and Supplementary Figure 1). The excess of heterozygosity and low recombination frequency distinctive in these genomic areas, together with the limited size of the population made it difficult to order these SNPs based on recombination events. Nevertheless, the length of the map (1,422 cM), the average distance between SNPs (1.75 cM) and the genome-wide recombination rate (2.16 cM/Mb) were similar to previous estimates using larger populations size (Table 3) (Mace *et al.* 2009; Bouchet *et al.* 2017).

**Table 3:** Comparison of two genetic linkage maps constructed with 108 recombinant inbred lines (RILs) derived from the cross between SC112-14 and PI609251 and a subset of RILs selected based on recombination events (*i.e.*, bin map)

		RILs (n = 108)		Bin Map (n = 31)		
Chromosome	SNPs^1^	Bin No.	Length (cM)	SNPs*^a^*	Bin No.	Length (cM)
1	579	149	217.55	573	94	277.00
2	466	100	151.35	466	55	153.00
3	316	79	147.24	315	56	175.25
4	479	89	147.89	473	47	128.26
5	378	85	129.79	376	57	187.22
6	420	86	140.60	419	56	179.57
7	247	58	122.60	247	39	133.34
8	287	58	115.29	287	46	164.55
9	390	74	129.83	390	44	122.98
10	276	66	120.77	276	43	122.33
Total	3838	844	1422.90	3822	537	1643.49

a Single nucleotide polymorphism identified by the genotype-by-sequencing analysis of the RIL population.

**Figure 1:**
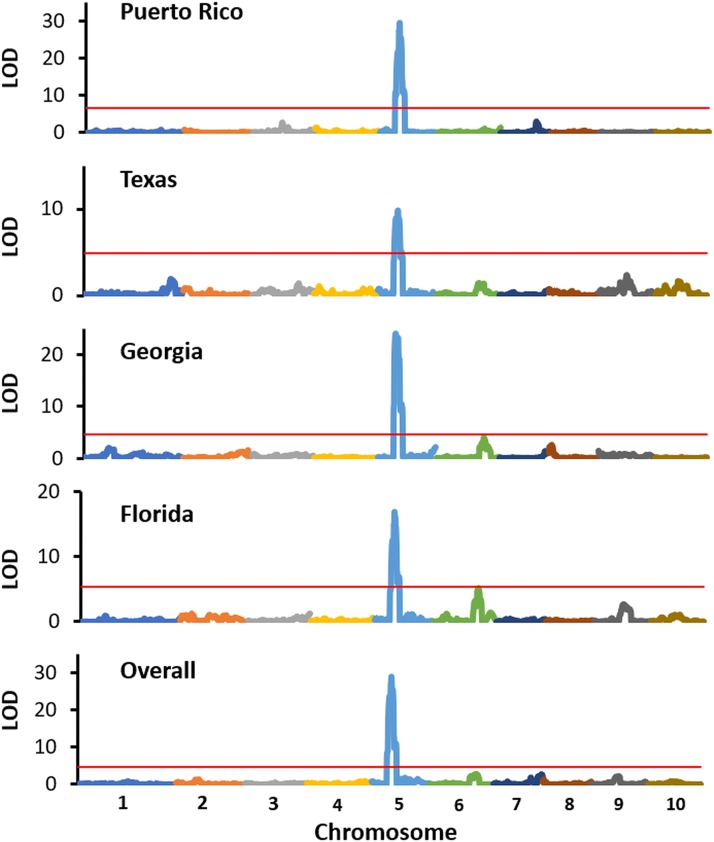
Composite interval mapping of anthracnose resistance response of 108 recombinant inbred lines (RILs) derived from the cross between SC112-14 and PI609251. Red horizontal lines mark an experiment-wise significance threshold of *P* <0.001 based on 1000 permutations.

A genome scan for anthracnose resistance response either across locations or within each location detected a 5.25 Mb region on chromosome 5 (62.34 - 67.59 Mb) that explains up to 59% of the observed variation (Table 4; Figure 1A). Another region of 1.18 Mb was detected on chromosome 6 (42.77 - 43.95 Mb) that explains 44% of the observed phenotypic variation in Florida. These two regions together explained most of the observed phenotypic variation in Florida. We observed that the association with the locus on chromosome 5 was stronger in Puerto Rico and Georgia (LOD = 29.56 and 23.97, respectively) than in Texas and Florida (LOD = 9.80 and 16.89, respectively).

**Table 4:** Genomic regions associated with the anthracnose resistance response reveled by the QTL analysis of the recombinant inbred line population derived from the cross between SC112-14 and PI609251 evaluated at four locations in 2016

Location	Chromosome	Region (Mbp)	LOD*^a^*	P.V.E*^b^*
Texas	5	64.19 - 65.20	9.80	23
Georgia	5	62.23 - 67.59	23.97	39
Florida	5	62.88 - 66.73	16.89	56
	6	42.75 - 42.78	5.18	44
Puerto Rico	5	62.35 - 67.57	29.56	59
Overall	5	62.34 - 67.59	28.98	30

a LOD values based on composite interval mapping as implemented in QTL Cartographer using a 3,838 SNPs linkage map.

b Percent of variance explained by the QTL.

To delimit these loci, we scrutinized the RILs to identify recombination events within the two genomic regions. Based on the analysis of 21 recombination events, the region on chromosome 5 was delimited to a 9.4 cM interval with a length of 1.58 Mb (65.02 – 66.60 Mb). This genomic region harbors 118 annotated genes (Sobic.005G170600 – Sobic.005G182900) with features shared by *R*-genes (*i.e.*, leucine-rich repeats, nucleotide binding site). The limited number of recombination events and smaller phenotypic effect of the region on chromosome 6 prevented us from further narrowing this region. This 6.2-cM interval harbored more than 82 genes (Sobic.06G067300 – Sobic.06G075400), none of which have previously been associated with anthracnose resistance.

### Bin mapping the resistance locus with 10 C. sublineolum pathotypes

The ten *C. sublineoulum* pathotypes exhibited different virulence against the subset of 31 RILs (Table 5; Figure 1B). Pathotype 29 (Texas) was the most virulent, affecting 14 RILs. In contrast, pathotypes 34 (Georgia) and AMP48 (Arkansas) both only affected five RILs. The field and greenhouse evaluations were correlated. Most of the RILs that scored <2.1 and >4.0 in field screening were resistant and susceptible, respectively, against the ten pathotypes. Remarkably, most of the RILs with a field score ranging from 2.3 to 4.0 exhibited both resistant or susceptible response against the ten pathotypes.

**Table 5:** Anthracnose resistance response of 31 recombinant inbred lines (RILs) derived from the cross between SC112-14 (P_1_) and PI609251 (P_2_) evaluated in the greenhouse against 10 different pathotypes from Texas (TX), Georgia (GA), Arkansas (AR) and Puerto Rico (PR)

		TX				PR		GA	AR		
RIL	Ant.*^a^*	Path.26	Path.20	Path.29	Path.31	Path.36	Path.32	Path.35	AMP 48	AMP50	AMP46
P_1_	2.00	R	R	R	R	R	R	R	R	R	R
P_2_	5.00	S	S	S	S	S	S	S	S	S	S
# 081	1.59	R	R	R	R/S	R	R	R	R	R	R
# 098	1.60	R	R/S	R	R	R	n.a.	R	R	R	R
# 039	2.00	n.a.	n.a.	n.a.	R	n.a.	R	n.a.	R	R	R
# 135	2.00	R	R	R	R	R	R	R	R	R	R
# 075	n.a.	R	R	R	R	R	R/S	R	R	R	R
# 130	n.a.	R	R/S	R/S	R/S	R/S	R/S	R	R	R/S	R
# 033	2.13	R	R/S	R/S	R/S	R	R	R	S	R	R
# 080	2.13	R	R	R	R	R	R	R	R	R	R
# 108	2.18	R/S	R/S	R/S	R/S	S	R	R	R/S	R	R
# 026	2.25	S	R/S	S	n.a.	R/S	R/S	R/S	R/S	R/S	n.a.
# 041	2.34	R	R	R	R	R	R	R	R	R/S	R
# 089	2.47	n.a.	R	R	R	R	R	R	R	R	R
# 035	2.63	R	R	R	R/S	R	R	R	R	R	R
# 088	2.88	R	S	R	R	R/S	R	R/S	R	R	R
# 125	2.93	n.a.	n.a.	R	S	n.a.	n.a.	n.a.	n.a.	S	n.a.
# 028	2.97	R/S	S	R/S	S	S	R/S	R/S	R/S	R/S	S
# 058	3.32	R	R	R	R/S	S	R	S	R	R	R
# 050	3.38	R/S	S	R	R/S	R	R	R	R	R/S	R
# 119	3.50	n.a.	R	n.a.	R	n.a.	n.a.	n.a.	R	n.a.	n.a.
# 002	4.00	S	S	S	S	S	S	S	S	S	S
# 027	4.03	R/S	S	R/S	R/S	R/S	R/S	R/S	S	S	S
# 086	4.06	R	R/S	R/S	R/S	R/S	R/S	R/S	R	R/S	R
# 034	4.13	R/S	S	S	S	S	S	S	R	R/S	S
# 008	4.38	S	S	S	S	S	S	S	R	R/S	S
# 009	4.38	S	S	S	S	S	S	R/S	R/S	S	S
# 127	4.50	S	S	S	S	S	S	R/S	R	S	S
# 104	4.60	R/S	S	R/S	S	R/S	S	R/S	R/S	R/S	R/S
# 123	4.63	R	S	R	R/S	R	R	R/S	R	R/S	R
# 122	n.a.	R/S	R/S	R/S	R/S	R	R/S	R	R/S	R/S	R/S
# 024	n.a.	S	S	S	S	S	S	R/S	S	S	S
# 025	n.a.	S	S	S	R/S	S	R/S	R/S	S	S	S

a Anthracnose resistance response in the field based on 1-5 scale (Prom *et al.* 2011), where 1-2 are considered resistant and 3-5 susceptible.

R and S refer to resistant and susceptible, respectively. R/S refer to segregation among the three replications.

A genome scan for anthracnose resistance response based on the bin mapping approach gave a similar result as an analysis of the whole population: both methods detected the 5.25 Mb region on chromosome 5 (62.34 - 67.59 Mb) (Table 6). This region was associated with the resistance response against nine pathotypes, whereas no genomic regions could be associated with resistance against pathotype AMP48. The observed associations were strongest for pathotypes AMP46 (-log p-value = 15.85) and Path.20 (-log p-value = 9.70) as a result of the variable resistance response observed among RILs with a field score ranging from 2.3 to 4.0. Moreover, we found that most of these RILs contained recombination events within the region that would have resulted in loss of resistance alleles, suggesting the whole region might be necessary to confer a broad resistance.

**Table 6:** Genomic regions associated with the anthracnose resistance response revealed by the association analysis of 31 recombinant inbred lines derived from the cross between SC112-14 and PI609251 evaluated against 10 *C. sublineolum* isolates under controlled conditions in the greenhouse

Origin	Pathotype	Chromosome	Region (Mbp)	-log p (*value*)*^a^*
Texas	Path.26	5	64.92 – 65.13	5.34
	Path.20	5	67.77 – 65.20	9.70
	Path.29	5	64.92	5.04
	Path.31	5	64.84 – 65.20	6.36
Puerto Rico	Path.36	5	64.77 – 65.02	5.85
	Path.32	5	64.84 – 65.19	6.31
Georgia	Path.35	5	64.19 – 65.20	7.72
Arkansas	AMP50	5	64.84 – 65.20	6.23
	AMP46	5	64.82 – 65.20	15.82
	AMP48	*no significant association detected*		

a -log p(value) based on the single marker analysis of 3,822 SNPs linkage map.

### Fine-mapping and genome-wide association analysis

**Figure 2:**
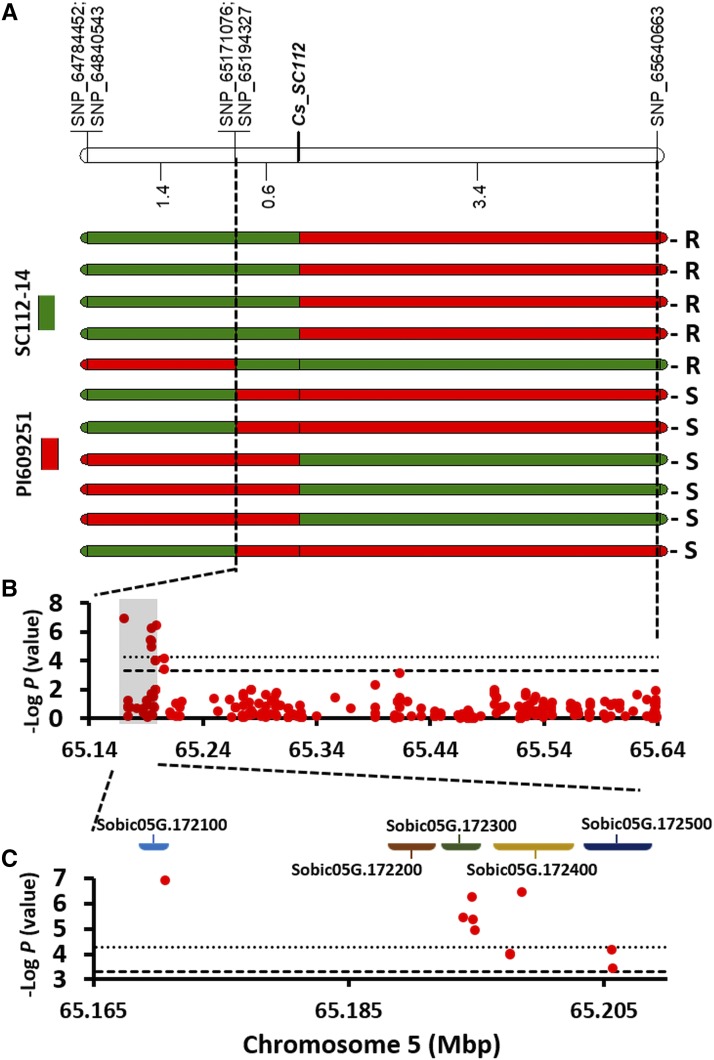
Genomic dissection of anthracnose resistance response in sorghum line SC112-14. A) Fine-mapping of the anthracnose resistance locus (*Cg_SC112*) on chromosome 5 based on the analysis of 1,500 segregating F_2:3_ progenies derived from crossing SC112-14 with PI609251. B) Manhattan plots for logistic regression based on a case-control analysis (*i.e.*, resistant or susceptible) of 574 accessions from the SAP and NPGS Ethiopian collection. Horizontal lines mark significance thresholds [*P* <0.05 (dashed) and *P* <0.001 (dotted)]. C) Delimited genomic regions and candidate genes in the *Cg_SC112* anthracnose resistance locus.

The analysis of 1,500 F_2:3_ segregating progenies identified 11 recombinants harboring the PI609251 allele (Figure 2A). Evaluation of anthracnose resistance response in the field revealed that five were resistant and six susceptible. The fragmentation of the locus into four segments showed that the 23-kb genomic interval showed the strongest association with the disease phenotype. In fact, it co-segregated almost perfectly with the resistance response, except in one heterozygous recombinant that exhibited a few anthracnose lesions. A genome-wide association study based on 574 genotypes (175 resistant and 399 susceptible) identified ten SNPs associated with anthracnose resistance (Figure 2B). Six SNP markers displaying statistically significant associations with the disease phenotype (-log p-value >4.94) were located within or near the 23-kb genomic interval (425 bp and 4 kb downstream and upstream, respectively), whereas the most distant SNP marker was located 11 kb downstream. This 34-kb interval contains five genes (Sobic.05G172100 to Sobic.05G172500) predicted to encode various protein domains, including Ankyrin repeats, F-box, plastid lipid-associated (PAP) and WD40 repeats (Figure 2C).

## Discussion

The utilization of multiple resistance sources is the most reliable alternative to control anthracnose disease in sorghum in the U.S. Today, the resistance response in sorghum lines Bk7 (Felderhoff *et al.* 2016), SC748-5 (Ramasamy *et al.* 2009; Burrell *et al.* 2015), SC414-12E (Patil *et al.* 2017), SC155-14E (Patil *et al.* 2017) and SC112-14 (Cuevas *et al.* 2014a) has been associated with genomic regions on chromosomes 9 and 5. However, these family-based approaches identified extended genomic regions that overlapped (*e.g.*, Bk7 and SC155-14E; SC414-12E and SC112-14), making it difficult to determine if they actually represent different resistance loci. For instance, based on previous association studies the region on chromosome 5 is associated with the anthracnose resistance response in sorghum line SC414-12E [64.02-66.98 Mb; Patil *et al.* (2017)] and resistant accessions in the SAP [65.19 - 66.49 Mb; Cuevas *et al.* (2018)], suggesting this region could harbor multiple sources of resistance. In this study, we delimited the anthracnose resistance response of sorghum differential line SC112-14 to a 34kb genomic region on chromosome 5 that harbors five candidate genes. Based on the screening at four locations and greenhouse against 10 *C. sublineolum* pathotypes, we confirmed this locus provides a broad resistance response that will be of value in breeding programs.

The involvement of other minor resistance genes could be necessary to confer a broad resistance response. A single dominant locus was described in the F_2_ generation used to develop the RILs (Cuevas *et al.* 2014a). However, the excess of susceptible lines observed in Puerto Rico, Georgia and Florida indicates the inbreeding increased the susceptibility to anthracnose. Since the excess of susceptibility is larger than expected based on the number of heterozygotes in the RIL populations, this susceptibility is likely associated with the lack of other complementary resistance alleles. Both the genetic map and the RIL population presented in this study have the power to dissect economically important traits into major-effect QTL, but the detection of minor-effect loci requires the use of a secondary mapping population in which previously detected major QTL are fixed (Sun *et al.* 2018). Hence, the introgression of the resistance from SC112-14 into commercial germplasm will need to involve the early fixation of the major QTL on chromosome 5 by marker-assisted selection followed by multi-location field screening for fixation of the other minor-effect loci.

Most disease resistance genes (*R*-genes) in plants encode proteins comprised of a variable amino-terminal domain, a central nucleotide-binding site (NBS) and a carboxy-terminal leucine-rich repeat (LRR) domain (Bent and Mackey 2007). Several genes within or in proximity to the 34 kb genomic region have been annotated to encode different types of transcription factors, precluding the identification of a single candidate gene. The definition of *R*-genes was recently modified due to the identification and characterization of numerous resistance genes with other functional domains. For instance, in Arabidopsis the gene *suppressor of nim1-1* (*SON1*) encodes a protein containing an F-box motif that provides resistance to both fungal and bacterial pathogens through the ubiquitin-proteasome pathway (Kim and Delaney 2002). The ankyrin (ANK) domain-containing proteins are involved in several physiological and developmental processes and are encoded by a large multi-gene family. For example, in rice and potato (*Solanum tuberosum* L.), ANK proteins play an important role in defense by regulating the salicylic acid and jasmonic acid pathways (Wu *et al.* 2009; Mou *et al.* 2013). The nature of these candidate genes suggests the resistance response in SC112-14 is based on signaling cascades and transcriptional reprograming, rather than recognition of pathotype-associated molecular patterns. In fact, the 34-kb genomic interval may play a role in the resistance response. Transcriptional analysis is expected to provide insights into the expression of these five candidate genes in response to *C. sublineolum*.

Fungal effectors are proteins that alter the structure or modulate the function of host cells to facilitate infection (Ellis *et al.* 2009). In some cases, an effector acts as an avirulence (*Avr*) factor that the resistant plant recognizes and responds to by activating their defense systems. Little is known about the diversity of *Avr* factors or the existence of multiple pathotypes in *C. sublineolum*. Greenhouse screening revealed that some RILs that were resistant or susceptible in the field could exhibit contrasting responses when challenged by a single pathotype. These results indicate that the variable anthracnose resistance response of some RILs in the field might be caused by the pathotype frequency variation. In fact, in the greenhouse RIL123 exhibited a resistance response against six pathotypes, but it was highly susceptible in field screening (score = 4.60). Therefore, greenhouse screening for anthracnose resistance response must include an adequate genetic representation of *C. sublineolum* pathotypes. Recent evidence suggests that the recognition of *Avr* factors could induce a resistance response against other related pathogens or races (Jones and Dangl 2006). Under field conditions, a given RIL faces constant and multiple *C. sublineolum Avr* factors that might activate the plant defense system, which leads to a broader resistance response against other pathotypes. Likewise, these *Avr* factors might activate different defense pathways, which would make them attractive targets for providing a broader resistance response. Certainly, the single-pathotype evaluation of the RILs provided evidence that other not yet identified genomic regions play an important role against other pathotypes. In this regard, the development of a set of near-isogenic lines could provide a useful germplasm resource to study resistance response against specific pathotypes.

The utilization of different anthracnose resistance sources is still limited by the lack of inheritance data and molecular tools that increase and facilitate the selection during the breeding process. The conversion of GBS-derived polymorphisms into a SNP-marker platform for high- throughput genotyping is the first step for its application in breeding programs (Mammadov *et al.* 2012). In this study, we developed and validated four KASP markers tightly linked to the anthracnose resistance locus on chromosome 5 that can be used for marker-assisted selection. In this regard we developed four SNP markers located upstream (SNP_64784452, SNP_64840542, SNP_65171076, and SNP_65194327) and one downstream (SNP_65640662) of the QTL. The combination of SNP_65171076 or SNP_65194327 with SNP_656640662 is the most effective strategy to select resistant plants because of the low recombination frequencies between these markers and the resistance locus. Since this 500-kb region might contain unfavorable alleles at other loci, the use of SNP_65171076 or SNP_65194327, both tightly linked to the QTL (0.6 cM) could be favorable if selection at early plant developmental stages is followed by multiple-trait field screening. The KASP technology has several advantages compared to other genotyping assays, including its adaptability to be adapted to low-, medium- and high-throughput systems. Today, the introgression of the *Cs-SC112* locus into elite germplasm could be achieved with limited field evaluation and it could be pyramided with other resistance sources present in other genomic regions.

## Conclusion

Sorghum differential line SC112-14 is an important source of anthracnose resistance effective across several geographic locations. Genome mapping based on RIL populations and a high-density SNP linkage map revealed that this resistance response is controlled by a major QTL on chromosome 5. Based on the analysis of a large number of segregating progenies and GWAS, the locus was delimited to a 34-kb region harboring five candidate genes involved in signaling cascades and transcriptional reprograming. The evaluation of a strategically selected subset of 31 RILs in the greenhouse provided evidence that the field resistance response results from challenges by multiple pathotypes in each location. Therefore, it is important to use multiple pathotypes in greenhouse screenings. Four SNP-based markers tightly linked to the resistance locus were developed and validated for use in marker-assisted selection. This research lays the foundation for understanding the molecular mechanisms underlying the inheritance of anthracnose resistance in SC112-14 and provides high-throughput molecular markers necessary for its utilization in sorghum breeding programs.
